# A Case of Violent Distortion of the Foot, Occasioned by a Rotation of the Astragalus, in Consequence of a Fall, and Accompanied with a Laceration of the Integuments at the Outer Ancle, and Exposure of a Portion of the Fibula

**Published:** 1794

**Authors:** William Guy

**Affiliations:** Surgeon at Chichester.


V. *A Case of violent Distortion of the Foot, occasioned by a Rotation of the Astragalus, in consequence of a Fall, and accompanied with a Laceration of the Integuments at the outer Ancle, and Exposure of a Portion of the Fibula. Communicated in a Letter to* Dr. Simmons *by Mr.* William Guy, *Surgeon at Chichester.*

IF any apology be necessary for my wishing to see the following history inserted in the next Volume of Medical Facts and Observations, I apprehend it is furnished by the circumstance of two cases of the same kind having happened in this neighbourhood, in both of which amputation was judged necessary, and performed.

I was desired by a surgeon to visit a young gentleman who had received a severe hurt in the foot, in consequence of his horse having reared up and fallen on him; the injury appeared of fuch magnitude, that the furgeon had little doubt but that amputation would be neceffary. I faw the patient in about four hours after the accident, during the whole of which time he had fuffered excruciating pain; his left foot was wrenched round, fo as to defcribe a quarter of a circle, in which pofition it remained, refting (as the patient lay on his back) on the great toe, and forming a right angle with the inner fide of the tibia. The violent and fudden diftortion of the foot was fuch, that the integuments were torn over the outer ancle, leaving about two inches of the fibula expofed.

Though appearances were fo formidable, there was reafon to hope that the real injury fuftained by the parts concerned, was not anfwerable to them; a violent diftortion had happened, but there was no luxation; the aftragalus, and confequently the foot, had only undergone a rotation; and the laceration was caufed, not by any difplacement of the fibula, but by the integuments being violently ftretched and torn over the head of that bone.

Impreft with thefe ideas, we made a gentle extenfion of the foot, and replaced it without the leaſt difficulty in its natural ſituation; the integuments being thus relaxed, as readily returned over the head of the fibula, and the lips of the wound came nearly in contact; ſoft, eaſy dreſſings were applied, and the leg and foot were fomented with flannel cloths, wrung out of hot water, and laid on the limb, all motion being carefully avoided; ſome blood was drawn, an opiate adminiſtered, and the antiphlogiſtic regimen ſtrictly enjoined. In a very ſhort time the patient fell into a profound ſleep, in which ſtate he continued ſeveral hours; he awoke much refreſhed and tolerably eaſy; no conſiderable pain or ſwelling enſued; at the end of the month he was able to ride on horſeback, and with the aid of a ſtick could walk pretty well; and in leſs than three months after the accident he recovered the perfect uſe of the joint.

It will clearly appear to any one, on examining the ſkeleton, that the ligaments of the joint were not ſo much ſtretched in this caſe as in a luxation; and that the aſtragalus, when turned round as above deſcribed, is almoſt as well adapted to the cavity of the inferior extremity of the tibia, as when in its natural poſition; the head of the fibula covering the poſterior inſtead of the lateral part of the aſtragalus, and the the lower extremity of the inner ancle falling into a ſmall depreſſion of the anterior part of the ſame bone.

